# Numerical Solution for the Extrapolation Problem of Analytic Functions

**DOI:** 10.34133/2019/3903187

**Published:** 2019-05-28

**Authors:** Nikolaos P. Bakas

**Affiliations:** Intelligent Systems Lab & Civil Engineering Department, School of Architecture, Engineering, Land and Environmental Sciences, Neapolis University Pafos, 2 Danais Avenue, 8042 Paphos, Cyprus

## Abstract

In this work, a numerical solution for the extrapolation problem of a discrete set of* n* values of an unknown analytic function is developed. The proposed method is based on a novel numerical scheme for the rapid calculation of higher order derivatives, exhibiting high accuracy, with error magnitude of* O*(10^−100^) or less. A variety of integrated radial basis functions are utilized for the solution, as well as variable precision arithmetic for the calculations. Multiple alterations in the function's direction, with no curvature or periodicity information specified, are efficiently foreseen. Interestingly, the proposed procedure can be extended in multiple dimensions. The attained extrapolation spans are greater than two times the given domain length. The significance of the approximation errors is comprehensively analyzed and reported, for 5832 test cases.

## 1. Introduction

Prediction always differs from observation [1], and extrapolation remains an open challenge [2] or even a* hopelessly ill-conditioned* [3] problem, in a vast variety of scientific disciplines. It relies on numerical methods which attempt to predict the future, unknown values of a studied phenomenon, given a limited set of observations. Its importance is reflected in the Scopus database [4], by a search with the term extrapolation, yielding nearly 150 thousands of research items, in a wide range of scientific fields ([Supplementary-material supplementary-material-1]). The visual presentation of the corresponding keywords ([Supplementary-material supplementary-material-1]) in a bibliometric map [5] reveals that extrapolation is closely associated with* modeling, simulation, uncertainty, *and* interpolation*, which is the reciprocal problem to simulate the unknown mechanism which produced the given data, aiming to predict utilizing the constructed model. Contradictorily to interpolation and despite the significance of extrapolation and forecasting methods, they exhibit a decreasing pattern in literature since the early 1980s ([Supplementary-material supplementary-material-1]), indicating the futility to predict for extended time frames. The given data is often called* time-series* and their extrapolation* forecasting*, which usually depend on machine learning algorithms such as support vector machines [6–8] and artificial neural networks [2, 9, 10], which construct nonlinear regression models, to predict beyond the known domain. Prediction algorithms are useful in a wide range of scientific disciplines, such as earth sciences [7, 9], finance [8, 11], computer science [12, 13], and engineering [14–16]. Several methods have been proposed for time-series forecasting [2] and competitions regarding accuracy have been conducted, utilizing statistical [11, 17] and machine learning procedures [2, 10, 15]; however, the prediction horizon regards only a small percentage (~20-30%) of the given data extension. Similarly, for less uncertain problems as the extrapolation of curves defined by polynomials (splines) [18–20], extrapolation can cause unpredictable results, and their extension should be short [21]. Apparently, prediction procedures are essential for a vast variety of scientific fields, always based on numerical interpolation methods.

More specifically, in the case of analytic functions, where the given data follow an unknown rigor mathematical law, any extrapolation results are too weak to be interesting [3]. Previous theoretical works focus on the interpolation problem, such as the ability of neural networks with one hidden layer to approximate analytic functions [22], proofs regarding the degree of rational approximation of an analytic function [23], and analysis of the rate of decrease of the best approximations of analytic functions [24]. Accuracy and convergence techniques are demonstrated in [25], yet it is shown theoretically [26] that the approximation of analytic functions cannot converge exponentially. Approximation of analytic functions is investigated utilizing a variety of approximators [27–29], such as Hermite [30, 31] and Airy functions [32]. Computational works regard the approximation of a function [33] as well as its derivatives [34, 35], investigating the interpolation errors among the given nodal points. Such errors are higher than the approximation errors (Runge phenomenon) especially at the boundaries [33], affecting dramatically the extrapolation outside the given domain. Moreover, the round-off errors of computing machines follow some stochastic law [36] and recent works deal with the high impact of infinitesimal errors in the given data, utilizing extended arithmetic precision [37, 38]. Accordingly, the constitution of an accurate numerical method to approximate an analytic function and its derivatives, which is vital for the extrapolation problem, remains a challenge.

The purpose of this study was to provide a generic numerical solution for the extrapolation problem. It was attained for extended extrapolation horizons of even greater than 200% the given domain length if the set of data are derived from an unknown analytic function and their precision is high. The rationale of the proposed method adheres to the following three stages: (a) interpolation of the set of values (*f*_1_, *f*_2_,…, *f*_*N*_) at specified points (*x*_1_, *x*_2_,…, *x*_*N*_) using integrated radial basis functions (IRBFs), (b) computation of high order derivatives {*f*′, *f*′′, *f*^(3)^,…} at any point of the closed domain [*x*_1_, *x*_*N*_] with high accuracy based on a novel numerical scheme, and (c) successive application of the Taylor series formula to extrapolate *f* at points outside the given domain. The method is capable of interpolating within the given data with high precision, avoiding the Runge phenomenon at the boundaries [33], as well as of computing the higher order derivatives with remarkable accuracy, which are fundamental problems in numerous applications. The effects of the method's parameters on the prediction errors were extensively investigated by their analysis of variance (ANOVA) and feature extraction utilizing the Random Forest [39] method, for 5832 test cases. Illustrative examples of highly nonlinear analytic functions in two and three dimensions demonstrate the prediction extents attained by the proposed method.

## 2. Numerical Solution

### 2.1. Extrapolation of One Infinitesimal dx

**Figure 1:**
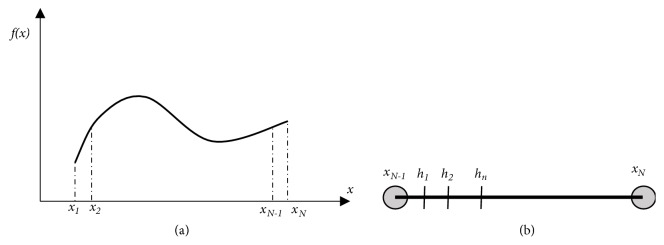
Given values of *f*(*x*) (a) and interpolation within the edge interval (b).

Let *f* be an analytic function, which is unknown. It is given that the function takes values **b** = (*f*_1_, *f*_2_,…, *f*_*N*_) at specified points **x** = (*x*_1_, *x*_2_,…, *x*_*N*_) as in Figure 1(a), for a generic analytic function. The extrapolation problem, that is, to predict the values which take the function outside the given domain [*x*_1_, *x*_*N*_], can be transformed into the computation of *n* ordinary derivatives of *f*, (*f*′, *f*′′,…, *f*^(*n*)^), at point *x*_*N*_. In particular, applying the Taylor formula from the point *x*_*N*_ to the next point *x*_*N*+1_ = *x*_*N*_ + *dx*, the corresponding value of *f* is given by

(1) fxN+dx=fxN+dxf′xN+dx22f′′xN+⋯+dxnn!fnxN,

for an infinitesimal step *dx*. *f*(*x*_*N*_ + *dx*) is the value of *f* at point *x*_*N*_ + *dx*, depending on the *n* ordinary derivatives of *f* at *x*_*N*_. These *n* derivatives should be computed, as they are unknown.

### 2.2. Numerical Evaluation of the Derivatives

Around the endpoint *x*_*N*_, we used the Taylor formula for computing the value of the function at the first predicted point *x*_*N*+1_. In order to calculate the derivatives, we need to consider the function values within the end interval *dx* from the point with abscissa *x*_*N*−1_ to *x*_*N*_. Accordingly, the Taylor expansion from the endpoint *x*_*N*_ to any previous point *h*_*j*_ → *x*_*N*_ (Figure 1(b)) may also be written in the form

(2) fxN−hj=fxN−hjf′xN+hj22f′′xN−⋯+−hjnn!fnxN.

By interchanging the positions of *f*(*x*_*N*_) and *f*(*x*_*N*_ − *h*_*j*_), we may write

(3) −fxN=−fxN−hj−hjf′hj+hj22f′′hj−⋯+−hjnn!fnhj,

and by applying it to *n* + 1 points *h*_*j*_  *within*  *the*  *end*  *interval dx* (Figure 1(b)) and writing the resulting system of equations in matrix form, we obtain

(4) $$\begin{array}{c} -f\left(\;x_{N}\;\right)\left[\begin{array}{c} 1\\ 1\\ \vdots \\ 1 \end{array}\right]=\left[\begin{array}{c} -f\left(x_{N}-h_{1}\right)-h_{1}f^{\prime }\left(x_{N}\right)+\frac{{h_{1}}^{2}}{2}f^{\prime \prime }\left(x_{N}\right)-\cdots +\frac{{\left(-h_{1}\right)}^{n}}{n!}f^{\left(n\right)}\left(x_{N}\right)\\ -f\left(x_{N}-h_{2}\right)-h_{2}f^{\prime }\left(x_{N}\right)+\frac{{h_{2}}^{2}}{2}f^{\prime \prime }\left(x_{N}\right)-\cdots +\frac{{\left(-h_{2}\right)}^{n}}{n!}f^{\left(n\right)}\left(x_{N}\right)\\ \vdots \\ -f\left(x_{N}-h_{n+1}\right)-h_{n+1}f^{\prime }\left(x_{N}\right)+\frac{{h_{n+1}}^{2}}{2}f^{\prime \prime }\left(x_{N}\right)-\cdots +\frac{{\left(-h_{n+1}\right)}^{n}}{n!}f^{\left(n\right)}\left(x_{N}\right) \end{array}\right]. \end{array}$$

It is important to note that all the derivatives are considered at the endpoint *x*_*N*_. Hence, extracting the derivatives as a column vector, we have

(5) $$\begin{array}{c} -f\left(\;x_{N}\;\right)\left[\begin{array}{c} 1\\ 1\\ \vdots \\ 1 \end{array}\right]=\left[\begin{array}{ccccc} -f\left(x_{N}-h_{1}\right) & -h_{1} & \frac{{h_{1}}^{2}}{2} & \cdots & \frac{{\left(-h_{1}\right)}^{n}}{n!}\\ -f\left(x_{N}-h_{2}\right) & -h_{2} & \frac{{h_{2}}^{2}}{2} & \cdots & \frac{{\left(-h_{2}\right)}^{n}}{n!}\\ \vdots & \vdots & \vdots & \ddots & \vdots \\ -f\left(x_{N}-h_{n+1}\right) & -h_{n+1} & \frac{{h_{n+1}}^{2}}{2} & \cdots & \frac{{\left(-h_{n+1}\right)}^{n}}{n!} \end{array}\right]\left[\begin{array}{c} 1\\ f^{\prime^{}}\left(x_{N}\right)\\ f^{\prime \prime }\left(x_{N}\right)\\ \vdots \\ f^{\left(n\right)}\left(x_{N}\right) \end{array}\right]. \end{array}$$

Thus, only the first column of the matrix in (5) varies as it refers to the values of* f* within the edge interval. As the objective is to compute the* n* ordinary derivatives of* f*, (5) is separated in submatrices, in order to rearrange the equations and develop a formula to calculate the vector** D**, containing the values of the* n* derivatives of* f* at point *x*_*N*_. The solid lines indicate the submatrices** B, C, H**,** D**, and **J**_*n*,1_; hence, (5) can be written in matrix partitions form

(6) $$\begin{array}{c} -f\left(\;x_{N}\;\right)\left[\begin{array}{c} 1\\ J_{n,1} \end{array}\right]=\left[\begin{array}{cc} A & B\\ C & H \end{array}\right]\left[\begin{array}{c} 1\\ D \end{array}\right], \end{array}$$

where

(6a) A=−fxN−h1,

(6b) B=h122⋯−h1nn!,

(6c) C=⋯−fxN−hn+1T,

(6d) H=h222⋯−h2nn!⋮⋮⋱⋮−hn+1hn+122⋯−hn+1nn!,

(6e) D=f′′xN⋯fnxNT,

(6f) Jn,1=⋯1T.

Since (6) is a system of *n* + 1 equations, in order to calculate the vector** D** (containing the values of the derivatives of *f* at point *x*_*N*_), by (6), we deduce

(7) $$\begin{array}{c} -f\left(\;x_{N}\;\right)J_{n,1}=HD+C, \end{array}$$

which is a system of* n* equations. Thus, the matrix of the* n *ordinary derivatives can be directly computed by

(8) $$\begin{array}{c} D=H^{-1}\left(\;-C-f\left(\;x_{N}\;\right)J_{n,1}\;\right), \end{array}$$

where* n*, and hence the number of the calculated derivatives, can be arbitrarily selected. Equation (8) offers a direct computation of the* n* ordinary derivatives** D**. Since** C** is still unknown, we can compute it, by interpolating* f* with IRBFs as defined subsequently.

### 2.3. Function Approximation with IRBFs

Radial Basis Functions (RBFs) networks are universal approximators while Integrated RBFs are capable of attaining precise approximation for a function and its derivatives [34, 35] as well as for the solution of partial differential equations [37, 38]. The essential formulation of RBFs, adopted in this work, is as follows.

Let *x* be a variable taking *N* values in a domain *X*. The known values of the function are given at the positions *x*_1_, *x*_2_,…, *x*_*N*_ as per Figure 1, while ∀*i* ∈ (1,2,…, *N* − 1) the interval length between two adjacent points *x*_*i*_, *x*_*i*+1_ is equal to *dx*. The given values *f*(*x*_*i*_) of the unknown function *f* can be approximated with RBFs *φ*(*x*), centered at the N points. The RBFs approximation is defined by

(9) $$\begin{array}{c} f\left(\;x_{i}\;\right)≅\overset{N}{\underset{j=1}{\sum }}a_{j}\varphi \left(\;r_{ij}\;\right), \end{array}$$

where *α*_*j*_ are the unknown weights for the approximation with RBFs, *r*_*ij*_ = |*x*_*i*_ − *x*_*j*_| represents the distance of a given point *x*_*i*_ in the domain from any other point *x*_*j*_, and *φ*(*r*_*ij*_) is the radial basis kernels such as Gaussian, Shifted Multiquadrics, and Sigmoid or their integrals, as in 14a, 14b, 14c, 14d, 14e, 14f. Applying (9) at the *N* given points results in the approximated values of *f*, *b*_*i*_ = *f*(*x*_*i*_); hence we deduce that

(10) $$\begin{array}{c} b_{i}=\overset{N}{\underset{j=1}{\sum }}a_{j}\varphi \left(\;r_{ij}\;\right). \end{array}$$

If we write (10) *N* times (*i* = 1,…, *N*) and define Φ as a matrix containing the values of *φ*(*r*_*ij*_), with* i* indicating the rows and* j* the columns of Φ, **α** = [*α*_1_, *α*_2_,…,*α*_*N*_]^*T*^ a vector containing the unknown approximation weights, and **b** = [*b*_1_, *b*_2_,…,*b*_*N*_]^*T*^ a vector comprising the values of *f* at the nodal points *x*_*N*_, we may write

(11) $$\begin{array}{c} b=\Phi \alpha . \end{array}$$

Hence we derive

(12) $$\begin{array}{c} \alpha =\Phi^{-1}b. \end{array}$$

The vector** b** is known, and the matrix Φ can be calculated from the distances of the known points *x*_*i*_ and *x*_*j*_, so the weights **α** of the RBFs approximation of the function *f* are computed by numerically inverting matrix Φ and multiplying it with vector** b**. After the computation of vectors **α**, the radial basis functions *φ*(*r*) can be recomputed for each intermediate point *h*_*j*_, in order to approximate the values of the unknown function *f* at these points. Accordingly, each approximation vector **φ**_*j*_ which corresponds to the points *h*_*j*_, **φ**_*j*_ = *φ*(|*x*_*i*_ − *h*_*j*_|), when multiplied with the calculated weights **α** of (12), results in the interpolated values *f*(*x*_*N*_ − *h*_*j*_) of 6c, and so

(13) $$\begin{array}{c} C={\left[\;\begin{array}{cccc} \varphi_{2} & \varphi_{3} & \cdots & \varphi_{n+1} \end{array}\;\right]}^{T}\alpha , \end{array}$$

where **φ**_*j*_ are the approximation vectors for each* h*_*j*_ and **α** the RBFs weights. The vectors **φ**_*j*_ are computed utilizing 14a, 14b, 14c, 14d, 14e, 14f, resulting in the vector** C**, which is the function's* f* approximation at points* h*_*j*_ (Figure 1(b)).

The definitions of the radial basis functions* φ* utilized in (12), (13) are specified in the following group of 14a, 14b, 14c, 14d, 14e, 14f: the Gaussian and the Shifted Logarithmic, as well as integrations of them, for one and two times. Equations 14a, 14b, 14c, 14d, 14e, 14f are written concerning *r* = |*x* − *x*_*j*_|, in order to be generic for any *x* ∈ [*x*_1_, *x*_*N*_] and hence for points* x*_*i*_ and* h*_*j*_.

The Gaussian RBF is defined by

(14a) $$\begin{array}{c} \varphi \left(\;r\;\right)=e^{-r^{2}/c^{2}}, \end{array}$$

where* c* is a constant influencing the shape of* φ *[40–42]. By integration of the Gaussian kernel, we obtain

(14b) $$\begin{array}{c} \varphi \left(\;r\;\right)=\int e^{-r^{2}/c^{2}}dx=\frac{c}{2}\sqrt{\pi }\mathrm{erf}\left(\;\frac{\left(x-x_{j}\right)}{c}\;\right), \end{array}$$

and if we integrate for a second time, then

(14c) φr=∫c2πerf⁡x−xjcdx=c2e−x−xj2/c2+cπx−xjerf⁡x−xj/c2,

where erf is the error function, $\mathrm{erf}\left(x\right)=\left(1/\sqrt{\pi }\right)\int_{-x}^{x}e^{-t^{2}}dt$, exhibiting a sigmoid scheme, which is commonly used in ANNs [43].

Respectively, the Shifted Logarithmic RBF is defined by

(14d) $$\begin{array}{c} \varphi \left(\;r\;\right)=\ln\left(\;r^{2}+c^{2}\;\right), \end{array}$$

and by integration of the kernel we obtain

(14e) φr∫ln⁡r2+c2dx=ln⁡x−xj2+c2x−xj−2x+2catan⁡x−xjc,

and if we integrate for a second time, then

(14f) φr=∫lnx−xj2+c2x−xj−2x+2c tan−1⁡x−xjcdx=xxj−x−xjc2+x2+xj2ln⁡x−xj2+c2−3x22+2cx−xjtan−1⁡x−xjc−xxj·ln⁡x−xj2+c2.

### 2.4. Iterative Implementation of Taylor Series Expansion

Exploiting the computed derivatives with high accuracy at the end of the domain *x*_*N*_, the predicted value of the function can be computed at the next step *x*_*N*+1_, by the Taylor series expansion of (1). This procedure is repeated iteratively, for each next point *x*_*N*+2_, *x*_*N*+3_,… utilizing each time the shifted vectors

(15a) b←fxN+dx,

(15b) x←xN+dx.

The* N* points are initially the positions where the function values are given. This procedure is iteratively applied using at each step the *N* − 1 previous values of the function (eliminating each time the first point) and one new value of the function, calculated by the procedure (15a and 15b). Keeping constant the length* dx* between a predicted point and its subsequent, the matrix Φ remains the same, as in the initial interpolation domain. The same stands for the inverted matrix Φ^−1^ as well as the matrices **φ**_*j*_, and despite the sequential shifts outside the original domain, the relative distances* r* of 14a, 14b, 14c, 14d, 14e, 14f remain the same. The same stands for the matrices** H **and** H**^-**1**^. Hence, the interpolation of the function within the end increment* dx* each time is accomplished by matrix multiplication and not inversion, which is performed only one time for Φ and one time for** H**. Utilizing this approach, during the iterations of the extrapolation procedure, the computational time is decreased dramatically, as these matrices are computed only once.

### 2.5. The Extrapolation Algorithm

In Algorithm 1, the procedure of extrapolating given values of* f* is demonstrated in algorithmic form, utilizing at each step the appropriate equations as formulated in Sections 2.1–2.4.

## 3. Results

### 3.1. Parameters Affecting the Method and Preparation of Dataset to Test Performance

The efficiency of the procedure was verified through numerical examples for a variety of highly nonlinear functions and their extrapolation spans. The extrapolation span is based on the accuracy of the interpolation method ((9), (10), (12), (13), 14a, 14b, 14c, 14d, 14e, 14f) as well as the numerical differentiation ((1), (5), (6), 6a, 6b, 6c, 6d, 6e, 6f, (8)). As demonstrated in Figure 1(b), the intervals for the differentiation* h*_*j*_ are selected near the *N* − 1^st^ node of the domain discretization, in order to achieve the highest possible accuracy, because this region is within the selected dx, and simultaneously, the interpolation error is minimized near this node, due to the Runge phenomenon [33]. Hence we select

(16) $$\begin{array}{c} h_{j}=dx-\frac{\left(j-1\right)dx}{10^{l}}, \end{array}$$

where* l *indicates the limit distance near the node *x*_*N*−1_. However, for extremely low values of* h*, the matrix** H **6d contains elements near zero, and its inversion grows into unstable one (high condition number and inversion errors).

The features which have an effect on the calculations, as well as their values (in parentheses) utilized in the parametric investigation, are the number of Taylor terms utilized, indicated as number of derivatives, (25, 50, 75); the number of computer digits used for the arbitrary precision calculations (500, 1000, 2000); the span of the given domain* L* (1/2, 1); the number of the divisions* N* (50, 100, 200); the limit* l* (5, 10, 20); the number of IRBFs integrations (0, 1, 2); the kernel of RBFs networks (Gaussian = 1, Shifted Logarithmic = 2); their shape parameter c (1/5, 1, 5); and the unknown function which is studied (sin⁡(x), e^cos(x)^). Accordingly, the resulting database consists of 5832 records of test cases. The output of each parametric execution of the proposed procedure was the condition number of the** H** and Φ matrices, their inversion errors, and the error of the first and second derivatives according to the proposed procedure. Moreover, the error of the first step of the extrapolation was computed (*ε*), in order to investigate its dependence on the problem statement parameters (*N, L, dx, f*) as well as the method's attributes (#derivatives, digits, kernel, c, #integrations).

### 3.2. Effects of Parameters on Extrapolation Accuracy and Computational Time

The *ε*′ = log_10_⁡(|*ε*| + 10^−323^) was utilized as a measure for the extrapolation error, as the values of *ε* exhibit a variation within the domain 4.623*∗*10^−283^ to the IEEE® arithmetic representation for positive infinity [44]. Given that the lower values of error *ε*′ are important, the analysis of variance was conducted for the cases with values of *ε* < 10^−50^; hence the resulting database consists of 2292 records. ANOVA found statistically significant differences between the means (MD) of *ε*′ for 25 and 50 derivatives (MD = 43.5803, p-value = 9.5606 *∗* 10^−10^) and for 25 and 75 derivatives (MD = 54.2206, p-value = 9.5606*∗*10^−10^) as demonstrated in [Supplementary-material supplementary-material-1] and [Supplementary-material supplementary-material-1]. Similarly, the number of digits ([Supplementary-material supplementary-material-1] and [Supplementary-material supplementary-material-1]) was found significant for the *ε*′ (MD = 20.3576, p-value = 9.6963 *∗* 10^−10^ for 500 to 1000 digits and MD = 33.9628, p-value = 9.5606 *∗* 10^−10^ for 500 to 2000 digits). The number of divisions exhibits a clear difference as well, in [Supplementary-material supplementary-material-1] and [Supplementary-material supplementary-material-1] (MD = 22.7727, p-value = 9.5606 *∗* 10^−10^ for 50 to 100 divisions and MD = 49.6978, p-value = 9.5606 *∗* 10^−10^ for 50 to 200 divisions). The univariate linear correlation of the condition number of Φ with *ε*′ ([Supplementary-material supplementary-material-1]) exhibits an R^2^ of 0.2559 (p-value = 3.1608 *∗* 10^−149^). For the condition number of** H **as well as the inversion errors of** H** and Φ, the correlations are even lower (Figures [Supplementary-material supplementary-material-1]-[Supplementary-material supplementary-material-1]); however, the majority of the values are 10^323^ for the condition of** H** and 10^−323^ for the inversion errors.

In order to further investigate more complex associations among the studied parameters and the extrapolation error *ε*′, the random forests method [39] was utilized. The numerical dataset was divided into a train set (85% of observations) and a test set (15%) in order to constitute and investigate the reliability of the predictive model. The R^2^ for the predicted versus actual *ε*′ for the test set was 0.8954 ([Supplementary-material supplementary-material-1]), indicating a reliable model. The features significance was evaluated in terms of their contribution to the prediction error in the constituted model ([Supplementary-material supplementary-material-1]), signifying high values for the studied function, the condition of Φ, the number of derivatives, and the domain length. Similarly, considering the computation time as dependent variable, the R^2^ for the test set was 0.9658 and the highest predictive features were the number of divisions, the number of integrations, and number of digits ([Supplementary-material supplementary-material-1]).

### 3.3. Illustrative Functions *f* and Their Extrapolation Spans

**Figure 2:**
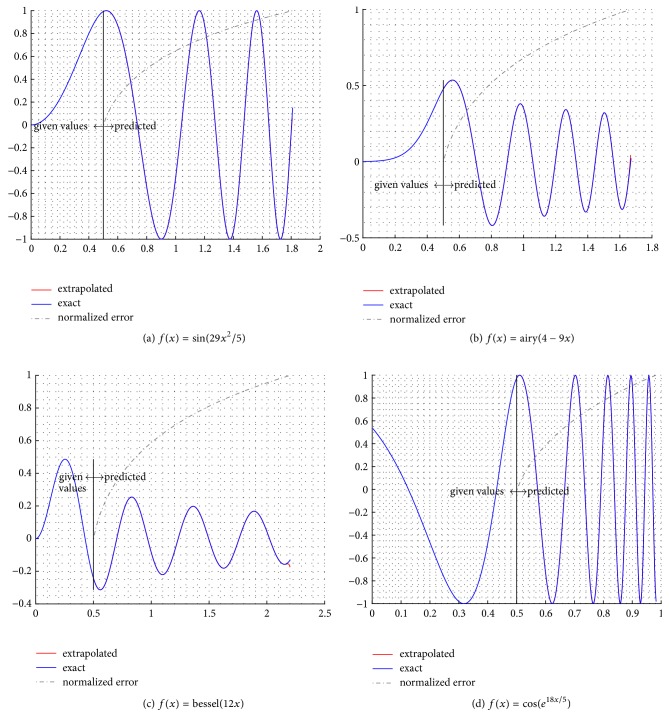
Extrapolation of analytic functions *f*(*x*). The prediction starts after the vertical line.

In Figure 2 the extrapolation results are demonstrated by four test functions as indicated. The initial domain contains only the known given data for *x* and *f*(*x*), and after the vertical lines, each graph contains the exact function values, the predicted and the normalized extrapolation error *ε*′′ = (log_10_⁡(|*ε*|) − min⁡(log_10_⁡(|*ε*|)))/(max⁡(log_10_⁡(|*ε*|)) − min⁡(log_10_⁡(|*ε*|))), which take values in the [0,1]. The text cases include a vast variety of analytic functions as well as their combinations. In Figures 2(a)–2(d) the given curvature or periodicity information is meager, compared to the predicted evolution of the function's values. Interestingly, the logged error plot exhibits a logarithmic scheme, indicating a weak form of stability [3].

**Figure 3:**
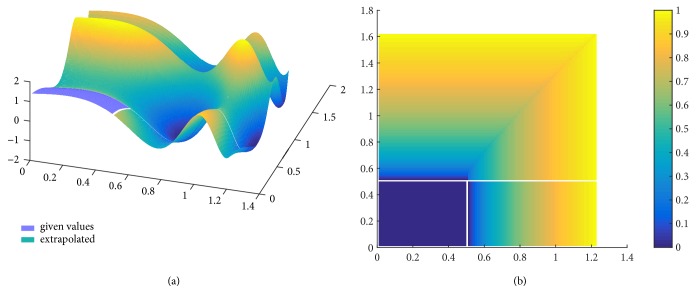
Extrapolation of function *f*(*x*, *y*) = sin(*e*^2*x*^) + cos(*e*^3*y*/2^) (a) and normalized errors (b).

In Figure 3, the proposed procedure is implied firstly in the* x*-axis and then in the* y*-axis for the function *f*(*x*, *y*) = sin(*e*^2*x*^) + cos(*e*^3*y*/2^). Given only the cyan region, the method can predict the highly nonlinear colored surface in the* x, y, z* space.

## 4. Discussion

### 4.1. Numerical Differentiation

Numerical differentiation is highly sensitive to noise [34], especially for the higher order derivatives. After an extensive examination the literature utilizing IRBFs [35, 45, 46] for the derivatives approximation, or for the solution of specific problems [47, 48] as well as other differentiation methods [49, 50] and a variety of formulations for IRBFs (14a, 14b, 14c, 14d, 14e, 14f), the proposed procedure accomplished striking accuracy, with error magnitude* ε* of* O*(10^−100^) or less (Figures [Supplementary-material supplementary-material-1]-[Supplementary-material supplementary-material-1] and supplementary database), for the derivatives' computation. This finally permitted the implementation of the Taylor method for significant extrapolation extents (Figures 2 and 3 and Data [Supplementary-material supplementary-material-1]). The numerical calculation of the derivatives with variable precision arithmetic offers high accuracy [37, 38]. The inverse problem of numerical integration exhibits lower errors with *ε* ~ *O*(10^−1000^); however, the integration is less sensitive to—even small—errors in the given data [51]. Cheng [37], similarly to this work, found that the errors of the derivatives approximation are of one or more order of magnitude higher than the function's (Figures [Supplementary-material supplementary-material-1], [Supplementary-material supplementary-material-1], and Data [Supplementary-material supplementary-material-1]). However, the digits studied were 100-200, while the precise computation of the derivatives was vital for the extrapolation, and so for a higher number of digits. Similarly, Mai-Duy et al. [52] examined the compactly integrated radial basis functions, with errors for the derivatives *ε* ~ *O*(10^−10^) for fifty digits accuracy.

### 4.2. RBFs' Matrix Φ and Shape Parameter

The approximation scheme ((9), (10), (12), (13), 14a, 14b, 14c, 14d, 14e, 14f) permits a precise interpolation, within the given domain, with *ε* ~ *O*(10^−100^) or less, as found in the numerical experiments (supplementary database). Accordingly, the approximation of the function's values is attainable with high accuracy, not only at the nodes* x*_*i*_, but for any intermediate point between two given points and, hence, for points* h*_*j*_ (Figure 1(b)), permitting the precise calculation of vector** C** (13). Arbitrary precision found capable of avoiding the ill-conditioning of Φ was also indicated by Cheng [37] and Huang [38]. Using arbitrary precision in the calculations, the inversion errors in ΦΦ^−1^**-I** were found of* O*(<10^−100^) or even exact zero (supplementary database). The arbitrary precision has also been used for the robust computation of Φ by Cheng [37], where the Gaussian RBFs and inversed multiquadrics exhibit accuracy of interpolation with *ε* ~ *O*(~10^−20^-10^−30^).

The association of the condition number of Φ with *ε*′ exhibits low R^2^ (0.25595) with slightly negative slope ([Supplementary-material supplementary-material-1]), although the high condition number is considered to increase instability [37, 41, 52] indicating the need for high precision to the calculations; however the majority of the values are 10^323^, that is, the maximum real number considered by the software [44]. Sensitivity analysis was selected instead of optimizing each method's parameters such as times of RBFs integration, the kernel function, or its shape parameter [40–42], as the interpolation errors in a number of the test cases were equal to zero at the nodes, eliminating any relevant objective function. The number of integrations of RBF* φ* increased computational time, as it magnifies the Φ and Φ^−1^ matrices, due to the more complex formulas (14b, 14c, 14e, 14f).

### 4.3. Summary of Findings and Limitations of the Proposed Solution

In brief, a method for the extrapolation of analytic functions is developed, accompanied by a systematic investigation of the involved parameters. The proposed scheme for the numerical differentiation exhibited low enough errors to permit adequate extrapolation spans. The constituted database of the numerical experiments highlights the fact that, for the same problem formulation (*L, N, dx, f*), the derivatives calculation exhibits a high variation, indicating the vagueness of the transference from the presumed theory to the actual calculations. The numerical investigation of the extrapolation errors suggests that only utilizing high accuracy and a precise approximation scheme for a function as well as its derivatives, the extrapolation is attainable. Thus, for real-world phenomena, with laboratory accuracies even of* O*(10^−20^), the predictions are limited to short length. Only if the measurements contain some hundreds of significant digits, the proposed solution is efficient. As this is difficult to be accomplished by laboratory instruments, this work's findings provide strong evidence that we are far from lengthy predictions in physics, biology, and engineering and also even more far from phenomena studied in health and social sciences. However, the parametric investigation suggests that the precision in calculations and the utilized methods are vastly significant, as the extrapolation horizons achieved by the proposed numerical scheme are about an order of magnitude higher than those in the existing literature, highlighting the potentiality of predictions.

## 5. Materials and Methods

All the software operations ran on an Intel i7-6700 CPU @3.40GHz with 32GB memory and SSD hard disk, and the execution time was accordingly tracked. The errors of the derivatives in the relevant literature are reported in the Supplementary Data [Supplementary-material supplementary-material-1], and they highlight the immense accuracy of numerical differentiation achieved in this work.

## Figures and Tables

**Algorithm 1:**
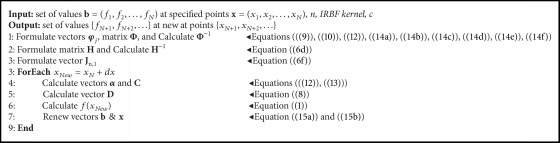
Extrapolating given data of an unknown function.

## Supplementary Materials

Supplementary Materials The supplementary materials are divided into two parts: (A) The supplementary figures and tables for the statistical analyses of parameters affecting extrapolation accuracy, and the bibliometric review figures. (B) The supplementary database: Data File S1. experiments.xlsx (attached): database for the parametric investigation of extrapolation errors. Figure S1: bibliometric map of papers with keyword extrapolation. Utilizing a bibliometric procedure [5] for a co-keywords based bibliometric analysis for the top 2000 papers in terms of their citations, containing extrapolation as keyword, the keywords highly associated with extrapolation—closer on the bibliometric map—are interpolation, risk assessment, prediction, uncertainty, modeling, etc. The almost coincident positions of extrapolation and interpolation indicate that the techniques aiming to extend a series of values beyond the observed domain are mainly based on interpolation procedures, which is an effort to mathematically formulate the phenomenon [53]. Figure S2: Evolution of terms in Google Books. Commencing the eighties and on, the effort of the researchers is decreasing, reflecting the failure to predict time-series values. This is demonstrated through a search in over 25 million—as of October 2015—books [54], for the words forecasting and extrapolation and the closely related topic of interpolation [53]. Figure S3: variance of *ε*′ for number of derivatives. Figure S4: variance of *ε*′ for number of digits. Figure S5: variance of *ε*′ for number of divisions. Figure S6: variance of *ε*′ for number of integrations. Nonsignificant differences (as per Table S5). Figure S7: variance of *ε*′ for domain length. Nonsignificant differences (as per Table S6). Figure S8: variance of *ε*′ for function. The function sin(*x*) exhibits less errors than the more complex e^cos⁡(x)^ (MD = 15.6422, p-value = 1.0597 *∗* 10^−10^, from Table S7). Figure S9: variance of *ε*′ for kernel. The Gaussian kernel exhibits lower errors than the Shifted Logarithmic (MD = 9.9051, p-value = 3.3708 *∗* 10^−7^ - Table S8). Figure S10: variance of *ε*′ for limit *l*. Nonsignificant differences (as per Table S9). Figure S11: variance of *ε*′ for shape parameter. The shape parameter 0.2 exhibits higher errors than 5 (MD = 8.5855, p-value = 0.0065 - Table S10). Figure S12: scatter plot for condition number of Φ and *ε*′. Figure S13: scatter plot for condition number of **H** and *ε*′. Figure S14: scatter plot for inversion error of Φ and *ε*′. Figure S15: scatter plot for inversion error of **H** and *ε*′. Figure S16: scatter plot for error of first derivative and *ε*′. Figure S17: scatter plot for error of second derivative and *ε*′. Figure S18: scatter plot for actual versus predicted *ε*′. Table S2: differences between the means of *ε*′ for groups with an uneven number of derivatives. Table S3: differences between the means of *ε*′ for groups with an uneven number of digits. Table S4: differences between the means of *ε*′ for groups with an uneven number of divisions. Table S5: differences between the means of *ε*′ for groups with an uneven number of integrations. Table S6: differences between the means of *ε*′ for groups with uneven domain lengths. Table S7: differences between the means of *ε*′ for groups with uneven functions* f*. Table S8: differences between the means of *ε*′ for groups with uneven RBF kernel. Table S9: differences between the means of *ε*′ for groups with uneven limit* l*. Table S10: differences between the means of *ε*′ for groups with uneven RBFs shape parameter. Figure S19: features significance.
