# Supplementary material for: Numerical Solution for the Extrapolation Problem of Analytic Functions
Source: Research (Wash D C). 2019 May 28;2019:3903187. doi: 10.34133/2019/3903187 (PMC6750076; doi:10.34133/2019/3903187)
Supplement: Supplementary Materials — The supplementary materials are divided into two parts: (A) The supplementary figures and tables for the statistical analyses of parameters affecting extrapolation accuracy, and the bibliometric review figures. (B) The supplementary database: Data File S1. experiments.xlsx (attached): database for the parametric investigation of extrapolation errors. Figure S1: bibliometric map of papers with keyword extrapolation. Utilizing a bibliometric procedure [5] for a co-keywords based bibliometric analysis for the top 2000 papers in terms of their citations, containing extrapolation as keyword, the keywords highly associated with extrapolation—closer on the bibliometric map—are interpolation, risk assessment, prediction, uncertainty, modeling, etc. The almost coincident positions of extrapolation and interpolation indicate that the techniques aiming to extend a series of values beyond the observed domain are mainly based on interpolation procedures, which is an effort to mathematically formulate the phenomenon [53]. Figure S2: Evolution of terms in Google Books. Commencing the eighties and on, the effort of the researchers is decreasing, reflecting the failure to predict time-series values. This is demonstrated through a search in over 25 million—as of October 2015—books [54], for the words forecasting and extrapolation and the closely related topic of interpolation [53]. Figure S3: variance of ε′ for number of derivatives. Figure S4: variance of ε′ for number of digits. Figure S5: variance of ε′ for number of divisions. Figure S6: variance of ε′ for number of integrations. Nonsignificant differences (as per Table S5). Figure S7: variance of ε′ for domain length. Nonsignificant differences (as per Table S6). Figure S8: variance of ε′ for function. The function sin(x) exhibits less errors than the more complex ecos⁡(x) (MD = 15.6422, p-value = 1.0597 ∗ 10−10, from Table S7). Figure S9: variance of ε′ for kernel. The Gaussian kernel exhibits lower errors than the Shifted Loga [file 3903187.f1.zip › Supplementary Materials.pdf]

Supplementary Materials

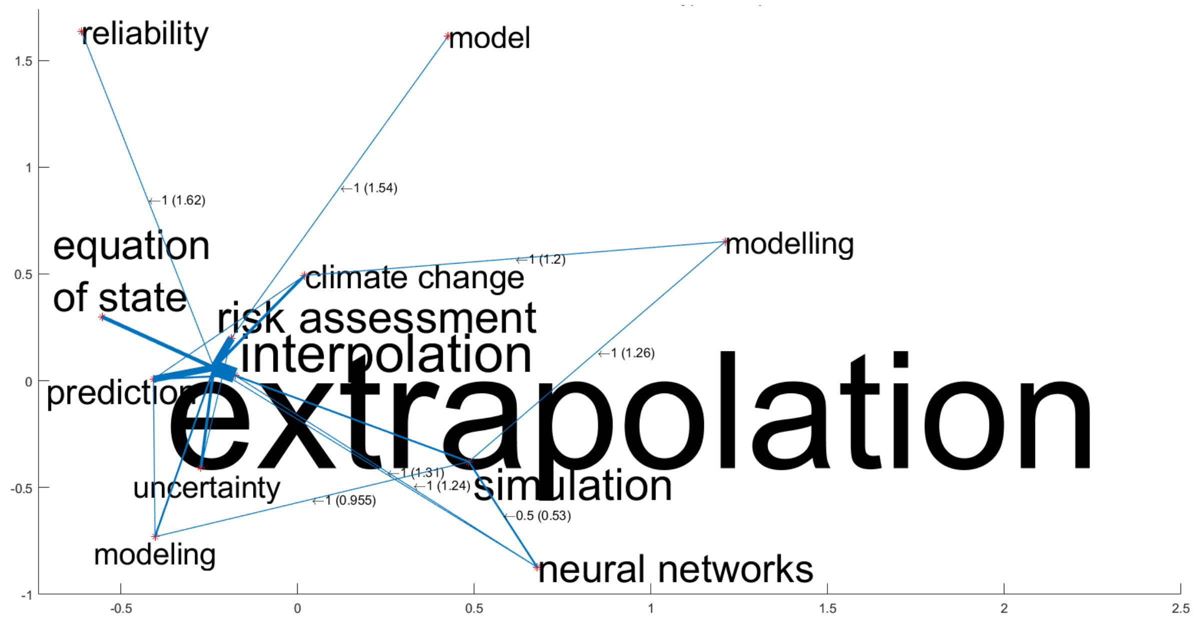

Figure S1: Bibliometric map of papers with keyword extrapolation. Utilizing a bibliometric procedure [5] for a co-keywords based bibliometric analysis for the top 2000 papers in terms of their citations, containing extrapolation as keyword, yield the keywords highly associated to extrapolation –closer on the bibliometric map- are: interpolation, risk assessment, prediction, uncertainty, modeling etc. The almost coincident positions of extrapolation and interpolation indicate that the techniques aiming to extend a series of values beyond the observed domain are mainly based on interpolation procedures, which is an effort to mathematically formulate the phenomenon [53].

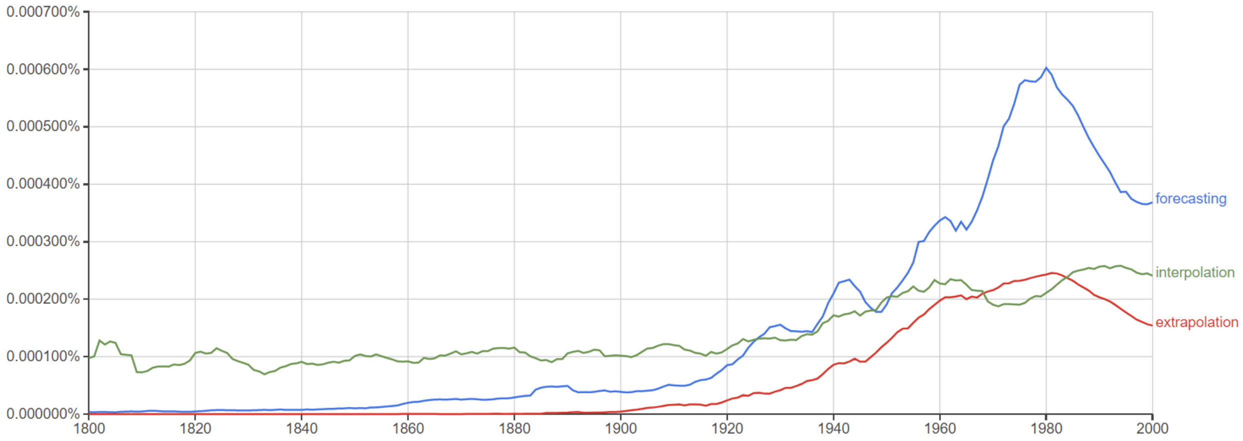

Figure S2: Evolution of terms in Google Books. Commencing the eighties and on the effort of the researchers is decreasing, reflecting the failure to predict time-series values. This is demonstrated through a search in over 25 million -as of October 2015- books [54], for the words forecasting, extrapolation and the closely related topic of interpolation [53].

Table S1: Number of Research Items (RI) containing the term extrapolation per subject area

Subject Area	RI	Subject Area	RI	Subject Area	RI
Physics and Astronomy	36,257	Computer Science	13,490	Arts and Humanities	2,239
Engineering	30,262	Agricultural and Biological Sciences	10,832	Decision Sciences	1,909
Medicine	19,539	Pharmacology, Toxicology and Pharmaceutics	9,538	Immunology and Microbiology	1,777
Chemistry	19,252	Chemical Engineering	6,403	Psychology	1,738
Earth and Planetary Sciences	17,980	Energy	5,205	Health Professions	1,610
Environmental Science	15,056	Social Sciences	3,949	Multidisciplinary	1,233
Materials Science	14,716	Business, Management and Accounting	2,637	Nursing	496
Mathematics	14,435	Economics, Econometrics and Finance	2,509	Veterinary	467
Biochemistry, Genetics and Molecular Biology	14,412	Neuroscience	2,382	Dentistry	179

Verification examples (supplementary figures and tables)

Effect size figures

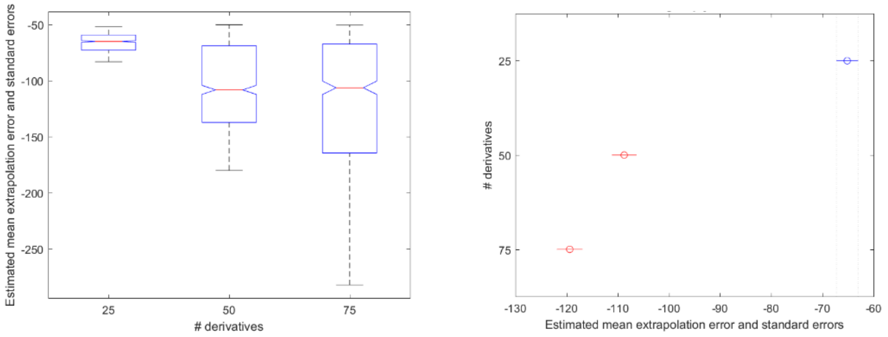

Figure S3: Variance of ε' for number of derivatives

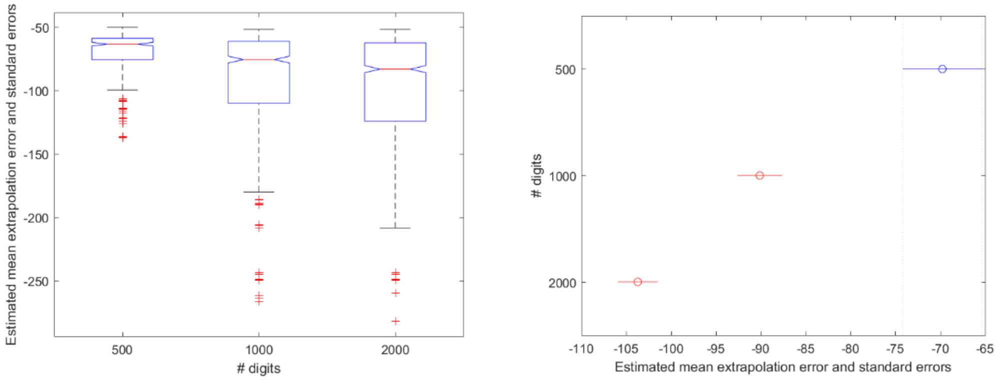

Figure S4: Variance of ε' for number of digits

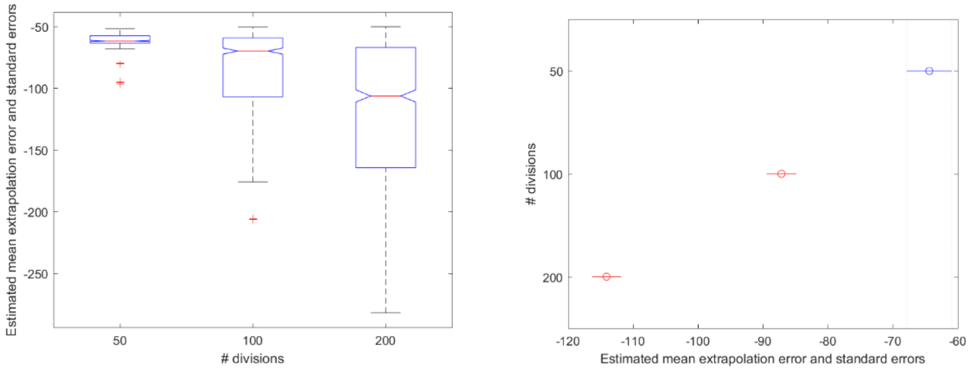

Figure S5: Variance of ε' for number of divisions

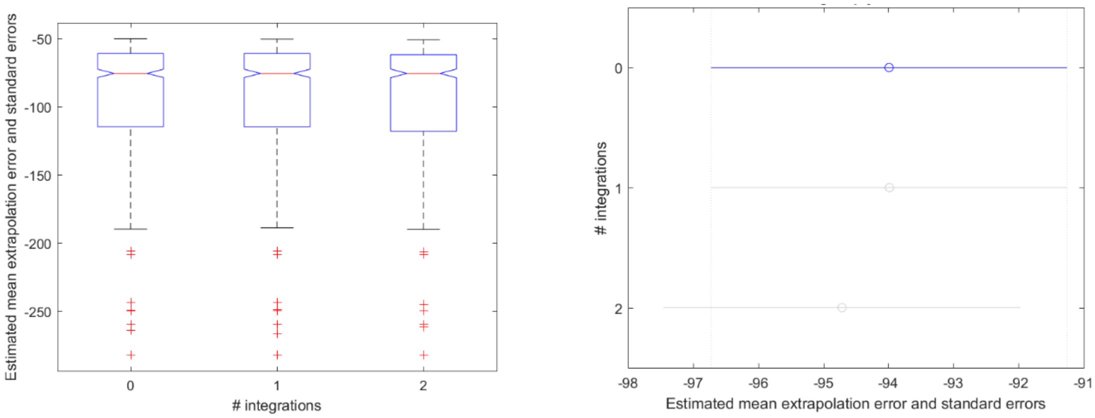

Figure S6: Variance of ε' for number of integrations Non-significant differences (as per Table S5).

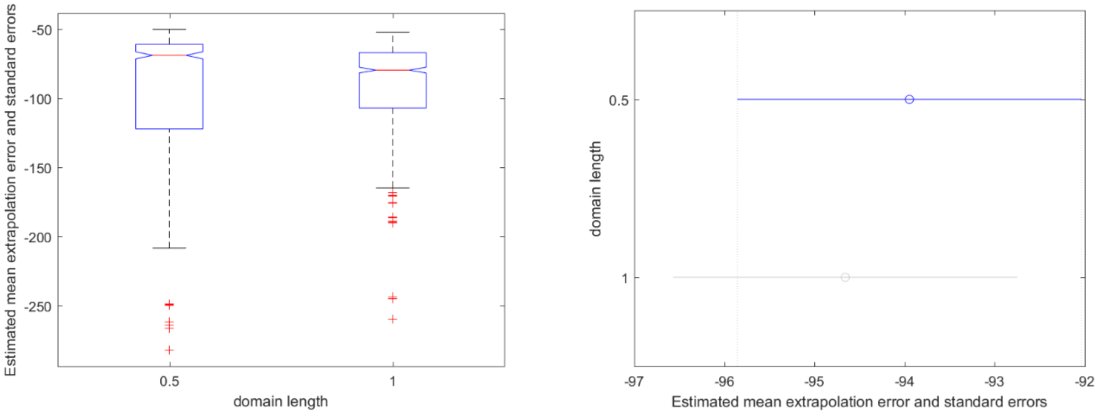

Figure S7: Variance of ε' for domain length. Non-significant differences (as per Table S6).

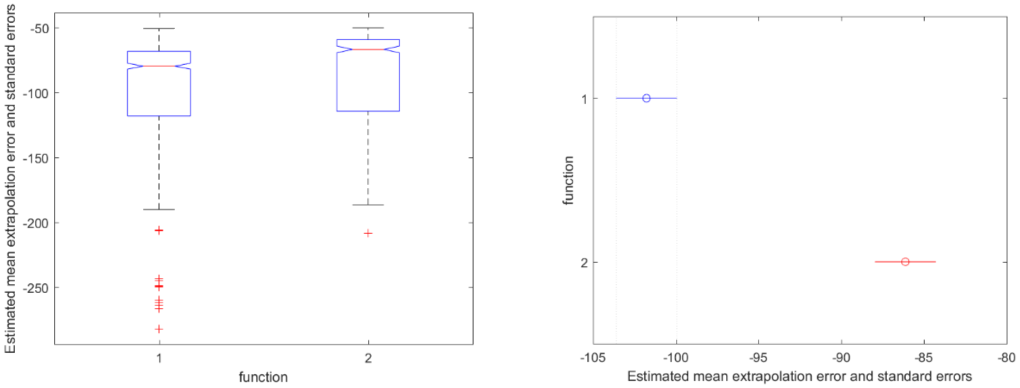

Figure S8: Variance of *ε'* for function. The function *sin(x)* exhibits less errors than the more complex $e^{\cos(x)}$ (MD=15.6422, p-value=$1.0597*10^{-10}$, from Table S7)

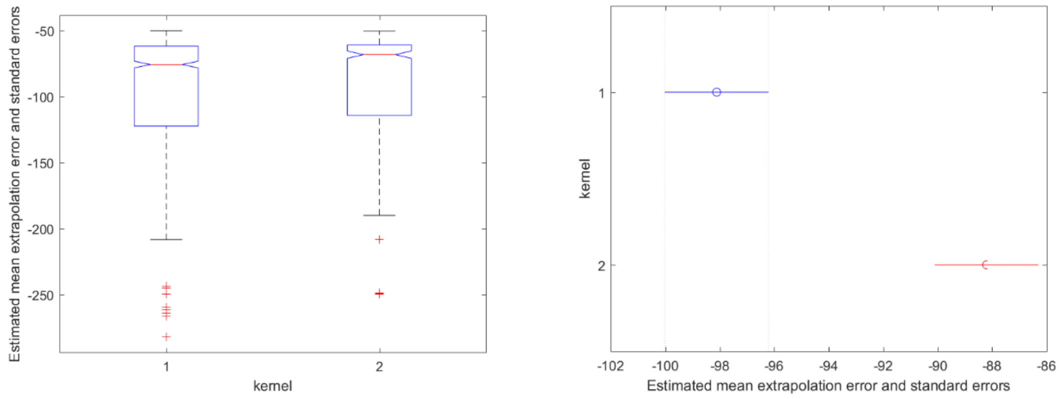

Figure S9: Variance of *ε'* for kernel. The Gaussian kernel exhibits lower errors than the shifted logarithmic (MD=9.9051, p-value=$3.3708*10^{-7}$ - Table S8)

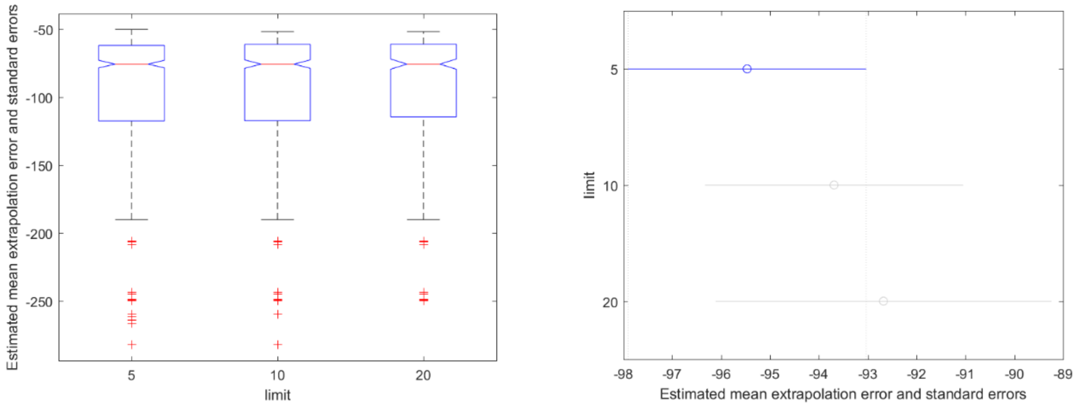

Figure S10: Variance of *ε'* for limit *l*. Non-significant differences (as per Table S9).

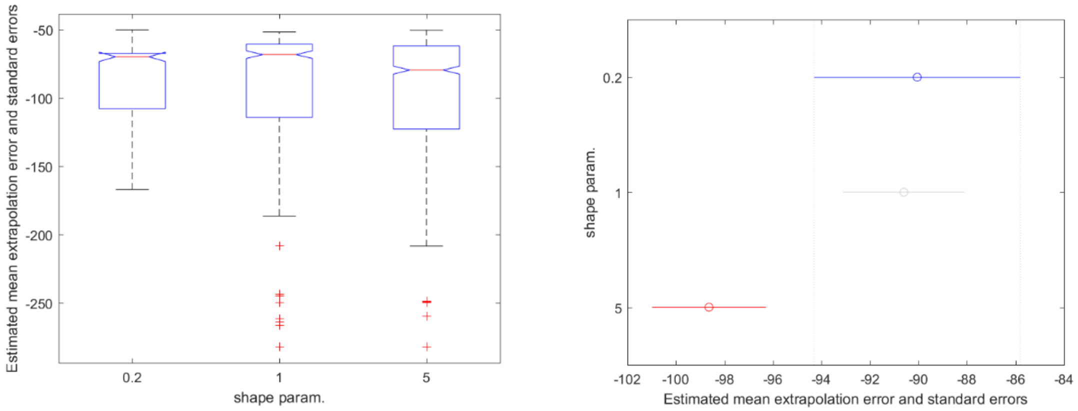

Figure S11: Variance of *ε'* for shape parameter. The shape parameter 0.2 exhibits higher errors than 5 (MD=8.5855, p-value=0.0065 - Table S10).

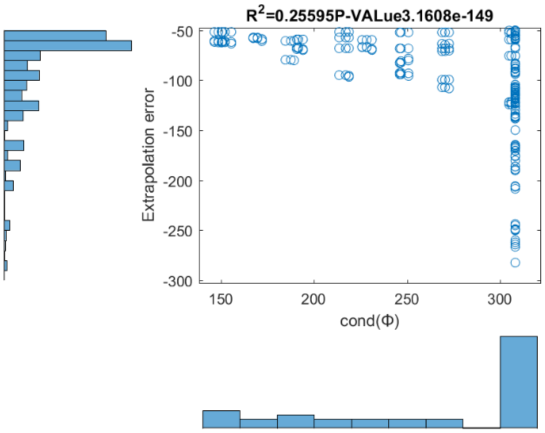

Figure S12: Scatter plot for condition number of **Φ** and *ε'*

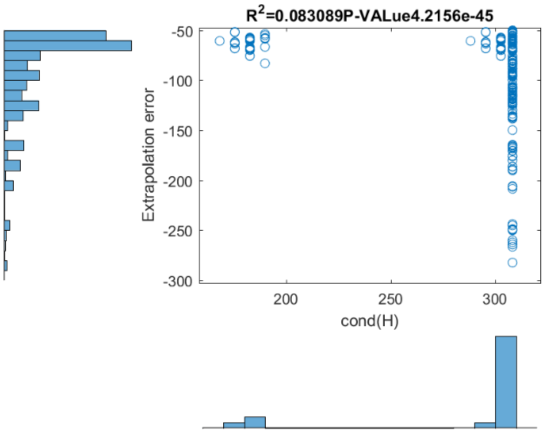

Figure S13: Scatter plot for condition number of **H** and *ε'*

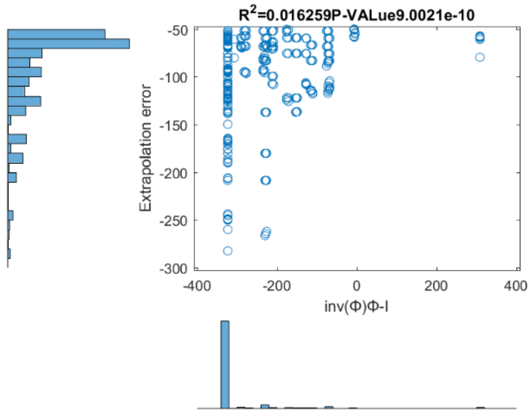

Figure S14: Scatter plot for inversion error of **Φ** and *ε'*

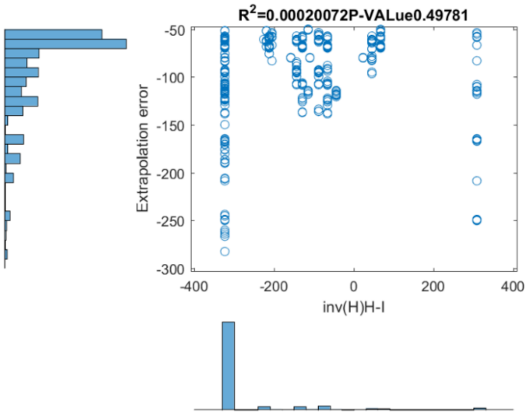

Figure S15: Scatter plot for inversion error of **H** and *ε'*

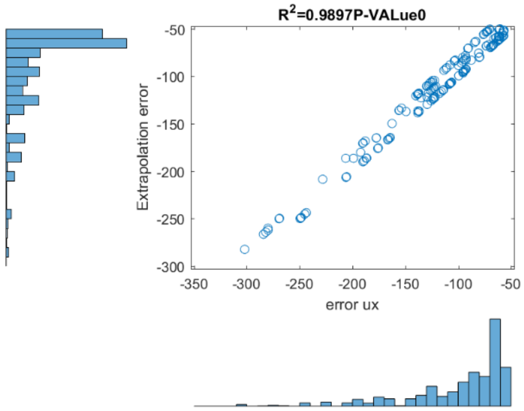

Figure S16: Scatter plot for error of first derivative and *ε'*.

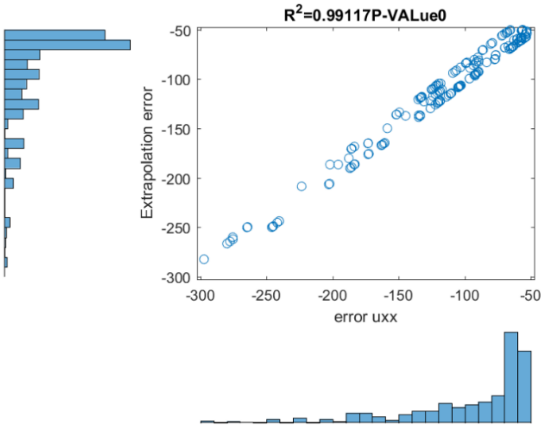

Figure S17: Scatter plot for error of second derivative and *ε'*

Interestingly, in Figs. S16 and S17, the errors of the first and second derivatives exhibit a clear linear correlation pattern with the error *ε'*, with p-values equal to zero, which further clarifies the importance of the derivatives computation and the contribution of the proposed procedure.

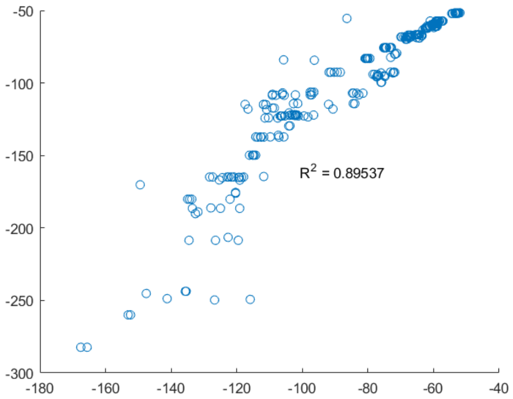

Figure S18: Scatter plot for actual versus predicted *ε'*

Effect size tables

The following Tables S2 – S10 present in the fourth column, the difference between the estimated group means, apropos the normalized error *ε'* (MD-ij- *ε'*). The corresponding compared Groups i & j are shown in the first and second columns. The third and fifth columns present the lower and upper 95% confidence intervals of the mean difference. Assuming the hypothesis that the mean difference among Groups i-j equals zero, the sixth column shows the corresponding p-value.

Table S2: Differences between the Means of *ε'* for groups with an uneven number of derivatives.

Number of derivatives					
Group i	Group j	-95%	MD-ij- *ε'*	+95%	p-value
25	50	39.0595	43.5803	48.1011	9.56e-10*
25	75	49.5921	54.2206	58.8491	9.56e-10*
50	75	5.7340	10.6402	15.5465	1.11e-06*

*significant at p<0.001.

Table S3: Differences between the Means of *ε'* for groups with an uneven number of digits

Number of digits					
Group i	Group j	-95%	MD-ij- *ε'*	+95%	p-value
500	1000	13.4553	20.3576	27.2599	9.70E-10*
500	2000	27.3314	33.9628	40.5943	9.56E-10*
1000	2000	8.8905	13.6053	18.3200	9.95E-10*

*significant at p<0.001.

Table S4: Differences between the Means of *ε'* for groups with an uneven number of divisions

Number of divisions					
Group i	Group j	-95%	MD-ij- *ε'*	+95%	p-value
50	100	17.0167	22.7727	28.5288	9.56E-10*
50	200	43.9835	49.6978	55.4121	9.56E-10*
100	200	22.4102	26.9250	31.4399	9.56E-10*

*significant at p<0.001.

Table S5: Differences between the Means of *ε'* for groups with an uneven number of integrations

Number of integrations					
Group i	Group j	-95%	MD-ij- *ε'*	+95%	p-value
0	1	-5.4758	-0.0052	5.4654	1.00E+00
0	2	-4.7529	0.7231	6.1991	9.49E-01
1	2	-4.7476	0.7283	6.2043	9.48E-01

Table S6: Differences between the Means of *ε'* for groups with uneven domain lengths

Domain length					
Group i	Group j	-95%	MD-ij- *ε'*	+95%	p-value
0.5	1	-3.1075	0.7121	4.5316	7.15E-01

Table S7: Differences between the Means of *ε'* for groups with uneven functions *f*

Function *f*					
Group i	Group j	-95%	MD-ij- *ε'*	+95%	p-value
1	2	-19.3264	-15.6422	-11.9581	1.06E-10*

*significant at p<0.001.

Table S8: Differences between the Means of *ε'* for groups with uneven RBF kernel

RBF kernel					
Group i	Group j	-95%	MD-ij- *ε'*	+95%	p-value
1	2	-13.7106	-9.9051	-6.0997	3.37E-07*

*significant at p<0.001.

Table S9: Differences between the Means of ε' for groups with uneven limit *l*

Limit *l*					
Group i	Group j	-95%	MD-ij- *ε'*	+95%	p-value
5	10	-6.8572	-1.7792	3.2989	6.90E-01
5	20	-8.6640	-2.7905	3.0830	5.06E-01
10	20	-7.0909	-1.0114	5.0681	9.20E-01

Table S10: Differences between the Means of ε' for groups with uneven RBFs shape parameter

Shape parameter					
Group i	Group j	-95%	MD-ij- *ε'*	+95%	p-value
0.2	1	-6.2006	0.5529	7.3063	9.80E-01
0.2	5	1.9859	8.5855	15.1851	6.50E-03*
1	5	3.1827	8.0326	12.8825	3.05E-04**

*significant at p<0.05; **significant at p<0.001.

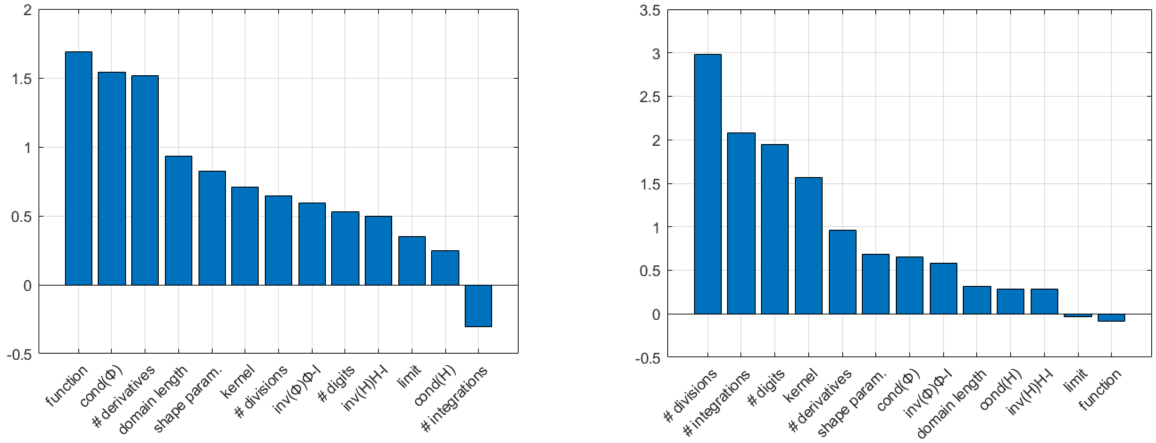

A: Extrapolation errors *B: Computation time*

Figure S19: Features significance

**Nomenclature**	
f	unknown analytic function to be extrapolated
N	number of given points
x	variable, the argument of f
dx	infinitesimal step equals the distances among given points as well as extrapolated
n	number of ordinary derivatives utilized for the solution
$f^{(n)}$	the nth ordinary derivative of f
h_j	distance of each interpolation point ($j=1,2,...,n$) within the end interval
**B**	row vector of dimensions *1* x *n*
**C**	column vector of dimensions *n* x *1*
**H**	matrix of dimensions *n* x *n*
**D**	column vector of dimensions *n* x *1*
$\mathbf{J}_{n,1}$	column vector of dimensions *n* x *1*
**α**	column vector of dimensions *N* x *1* containing the approximation weights
r	the distances among two points in the studied domain
φ	the radial basis function utilized for the solution
**x**	column vector of dimensions *N* x *1* containing the values of x
**b**	column vector of dimensions *N* x *1* containing the values of $f(x)$
**Φ**	matrix of dimensions *N* x *N*
c	constant influencing the shape of φ
